# KINLESSNESS AND COGNITIVE FUNCTION AMONG EUROPEAN OLDER ADULTS

**DOI:** 10.1093/geroni/igac059.1580

**Published:** 2022-12-20

**Authors:** Christine Mair, Katherine Ornstein

**Affiliations:** University of Maryland, Baltimore County (UMBC), Baltimore, Maryland, United States; Johns Hopkins, University, Baltimore, Maryland, United States

## Abstract

So-called “kinless” older adults (unpartnered and childless) are a growing population across the globe who may be at higher risk for social isolation and cognitive decline. Yet, this group of older adults is understudied in dementia-related research and their risk of cognitive decline likely varies by country context. We analyze data from the Survey of Health, Ageing & Retirement in Europe (SHARE) to explore cognitive decline by family structure and country. We assess cognitive decline along a gradient of “kinlessness” by comparing older adults with a) partner and child, b) partner and no child, c) no partner and child, and d) no partner and no child across 20 European countries. Results of this study will outline the dementia risks faced by the growing population of older adults with non-traditional family structures and highlight characteristics of country context that might enhance or reduce risk of cognitive decline for “kinless” older adults.

